# Genetic mapping of Fusarium wilt resistance in a wild banana *Musa acuminata* ssp. *malaccensis* accession

**DOI:** 10.1007/s00122-020-03677-y

**Published:** 2020-09-12

**Authors:** Fajarudin Ahmad, Nani M. Martawi, Yuyu S. Poerba, Hans de Jong, Henk Schouten, Gert H. J. Kema

**Affiliations:** 1 Research Center for Biology, Indonesian Institute of Sciences (LIPI), Jl. Raya Jakarta-Bogor Km. 46, Bogor, 16911 Indonesia; 2 Wageningen Plant Research, Wageningen University and Research, P.O. Box 16, 6700 AA Wageningen, The Netherlands; 3 Department Biology Education, Faculty of Education and Teacher Training, Universitas Sultan Ageng Tirtayasa, Kampus 2 Untirta, Jl Ciwaru Raya No. 25, Kota Serang, Banten Indonesia; 4 Laboratory of Genetics, Wageningen University and Research, P.O. Box 16, 6700 AA Wageningen, The Netherlands; 5 Department of Plant Breeding, Wageningen University and Research, P.O. Box 386, 6700 AJ Wageningen, The Netherlands; 6 Laboratory of Phytopathology, Wageningen University and Research, P.O. Box 16, 6700 AA Wageningen, The Netherlands

## Abstract

Banana is an important fruit and food crop, but is threatened by Fusarium wilt, one of the most devastating soil-borne fungal diseases. Only host resistance facilitates banana cultivation in infested soils around the world, but the genetic basis of Fusarium wilt of banana (FWB) is unknown. We selfed a heterozygous wild banana accession *Musa acuminata* ssp. *malaccensis* (Mam, AA, 2*n* = 22) to generate a mapping population and to investigate the inheritance of resistance to Race 1 and tropical race 4 (TR4) that cause FWB. Phenotyping (*N* = 217) revealed segregation for resistance, and genotyping by sequencing resulted in 2802 high-quality single-nucleotide polymorphic markers (SNPs) that were used for genetic mapping. Combined analyses of these data showed that a single dominant resistance locus controls resistance to Race 1 and maps near the distal part of chromosome 10. Recombinants, together with the position of the putative resistance gene, were further analysed using graphical genotyping, which retrieved markers flanking a 360 kb genetic region that associates with Race 1 resistance. The region contains 165 putative genes on the reference genome, including 19 leucine-rich repeat receptor-like kinase-like genes. At the same position and phase, we also identified a QTL for TR4 resistance, showing that the locus for resistance against Race 1 provided partial resistance to TR4. However, this effect was far less significant and hence not included in the mapping. These data support the breeding of new banana varieties with resistance to Fusarium wilt.

**Electronic supplementary material:**

The online version of this article (10.1007/s00122-020-03677-y) contains supplementary material, which is available to authorized users.

## Introduction

Fusarium wilt is the most devastating disease in banana culture and destroyed large plantations in the tropical countries of South America since the outbreak in the early 1900s (Ploetz 2005, 2015). The causal agents of the disease are a suite of *Fusarium* species (Maryani et al. 2019) that previously were classified as *Fusarium oxysporum* f. sp. *cubense* (Foc). They invade banana roots and subsequently colonize and occlude the vascular system which leads to severe wilting that eventually kills the plant (De Ascensao and Dubery 2000). According to the compatibility of the fungus with groups of banana cultivars, *Fusarium* strains are classified into three races. Race 1 is known for its devastation of large areas of ‘Gros Michel’ in 1950s. Race 2 is compatible with the cooking banana subgroup Bluggoe (ABB). The third type, Race 4, is usually divided into sub-tropical race 4 (STR4) that infects banana under abiotic stress and tropical race 4 (TR4), which devastates Cavendish plantations around the world, but also affects many other banana varieties (García-Bastidas 2019). TR4 was recently identified as a new species named *F. odoratissimum* (Maryani et al. 2019), which most likely originates from the Indonesian archipelago from where it disseminated globally (Maymon et al. 2020; Özarslandan and Akgül 2020; García-Bastidas et al. 2019a; Damodaran et al. 2019; Chittarath et al. 2018; Maymon et al. 2018; Ordoñez et al. 2015, Ordoñez et al. 2016; García-Bastidas et al. 2014).

*Fusarium* spp. causing Fusarium wilt of banana (FWB) are soil-borne fungi with the ability to produce chlamydospores. Such inoculum can survive for more than 30 years either as spores or by hiding as endophytes in weeds (Salacinas 2019), which makes disease control extremely challenging (Blomme et al. 2011; Ploetz 2005). Planting disease-free plantlets from in vitro culture may initially keep banana plants healthy, but after a few production cycles plants become affected and yields will dramatically drop due to massive fungal infestations (Bubici et al. 2019). Over the years, many products and conditions were trialled, including biocontrol applications with *Trichoderma* spp. or non-pathogenic *F. oxysporum*, in an attempt to control FWB (Dita et al. 2018; Chaves et al. 2016; Soluri 2005). However, none of these adequately managed the disease, except for the excelling resistance in Cavendish clones that were eventually embraced by the industry and quenched the Race 1 driven epidemic in ‘Gros Michel’ (Bubici et al. 2019).

Despite the success of Cavendish, the genetic basis of its resistance to Race 1 strains was never unveiled. Various wild and cultivated bananas were described conferring different levels of resistance to Race 1 and TR4 (García-Bastidas 2019). Interestingly, *Musa acuminata* ssp. *malaccensis* (Mam) ‘Pahang’, ssp. *burmannicoides* (Calcutta 4) and *M. itinerans* carry resistance to TR4 (D’Hont et al. 2012; Zuo et al. 2018; Zhang et al. 2018, 2019; García-Bastidas 2019), whereas the cultivar ‘Rose’ and ‘Tuu Gia’ (ITC 0610) are examples of resistant edible bananas (Houbin et al. 2004; Zuo et al. 2018; García-Bastidas 2019). However, despite their exquisite value, such germplasm remains largely untapped in contemporary breeding programs.

In recent studies, Peraza-Echeverria et al. (2008, 2009) used PCR-based analyses of RNA data to identify resistance gene analogues (RGAs) from two resistant and two susceptible accessions of Mam. They identified five RGAs of which three were associated with TR4 resistance. Eventually, *RGA2* was transformed to the susceptible Cavendish clone ‘’Grand Naine’’ which turned it resistant to TR4 (Dale et al. 2017). Actually, *RGA2* is present in Cavendish, but the gene is expressed 10 × lower than in the transgenic line, indicating that the resistance of the latter is due to a dosage effect.

Since resistance to Race 1 is an absolute prerequisite for any breeding program, we set out a strategy to identify the responsible genes, which enables marker-assisted breeding and avoids lengthy and expensive phenotyping assays. Genetic mapping is a powerful tool for identifying the locus of interest and the distances between genes on linkage groups. In banana, genetic maps have been constructed from segregation populations of selfed or cross-pollinated heterozygotes. Faure et al. (1993) built such a genetic map from an F_2_ population of two wild *M. acuminata* varieties based on RFLP, isozyme and RAPD markers, demonstrating 36% segregation distortion of the male-specific alleles. The linkage maps reported by Kayat et al. (2009) were based on two F_1_ populations of selfed Mam using AFLP, STMS and RAPD markers, resulting in 32 and 37 linkage groups, many more than the expected 11 groups of diploid banana (2*n* = 2*x* = 22). Kilian et al. (2012) generated a large number of markers by Diversity Array Technology (DArT) that were used by Hippolyte et al. (2010) in combination with SSR markers to build a linkage map from F_1_ progeny of a hybrid between *M. acuminata* ‘Borneo’ and ‘Pisang Lilin’ that resulted in the expected 11 linkage groups. Here, we developed a segregating population from selfing of a heterozygous Mam that was genotyped using the DArTseq genotyping-by-sequencing (GBS) method and was phenotyped for resistance to Race 1 or TR4. The phenotypic information was combined with SNP markers derived from the DArTseq analysis to build a linkage map. We subsequently mapped windows for Race 1 and TR4 resistance on chromosome 10. The identified flanking markers can now—for the first time—be used for marker-assisted breeding to FWB resistance.

## Materials and methods

### Creating a segregating population

A segregating population was made by selfing a diploid heterozygous accession of a wild *Musa acuminata* Colla var*. malaccensis* (Ridl.) Nasution (Mam) that originated from Sumatra, Indonesia. It was chosen for its resistance to TR4 in a greenhouse bioassay and in the field (data not shown). The selfing was performed at the Research Center for Biology, Indonesian Institute of Sciences (LIPI) in the field in 2014 by hand pollination. The pollinated flowers were bagged immediately using fine insect screens to prevent cross-pollination, and fruits were harvested 12–15 weeks after pollination. Subsequently, the seeds were collected in a week when the fruits were ripe. A total of 8077 seeds from 231 pollinated flowers was harvested from the selfed parent. The majority of the seed (90%) had black hard skins and were full of endosperm, while the remainder had brown or shrunken seed coats. The seeds were transferred to Wageningen University and Research (WUR) for embryo rescue and in vitro propagation. Prior to embryo rescue, the seeds were soaked in 96% ethanol for a minute and in 20% hypochlorite for 20 min, washed in sterile water, soaked in 10% hypochlorite for 10 min and finally washed in sterile water. Subsequently, we performed a priming by soaking the seeds in 10 ppm gibberellic acid (GA3) for three days to initiate shooting of the embryo (Arun et al., 2013). The embryos were taken out of the seeds under sterile conditions, transferred to Murashige & Skoog medium (MS) (Murashige & Skoog, 1962) with 2 ppm benzylaminopurine (BAP), 1 ppm biotin, 0.1 g/l myo-inositol and 3 g/l gelrite before autoclaving and placed in the dark to induce root elongation. After two to four weeks, the shooting embryos were exposed to light for shoot development. In total, 718 embryos were rescued, but also suffered from a bacterial contamination, whereas other embryos did not shoot (data not shown). Eventually, 255 embryos survived and developed into plants for disease assays by separating axillary shoots by subculturing on MS medium with 2 ppm BAP. Two to five times subcultures were taken to obtain 20–25 shoots per genotype. During the last subculturing, we transferred the shoots to MS medium without hormones to induce root formation. Subsequently, plants were transferred from tissue culture to the greenhouse in individual pots with soil (5% Swedish sphagnum peat, 41% grinding clay granules, 5% garden peat, 4% beam structure, 33% steamed compost and 12% PG-Mix 15-10-20) and maintained for 2 weeks under controlled conditions (100% humidity and 28 ± 2 °C) to acclimatize. Subsequently, the plants were kept at 75–85% humidity and 28 ± 2 °C for 2 months prior to inoculation.

### Disease assays

The disease tests were performed from August 2015 until October 2016, using a Race 1 isolate of unknown vegetative compatibility (Ordóñez, 2018) originating from Brazil (coded as Foc.CNPMF.R1) that was recovered from ‘Maçã’ banana (ABB, Silk subgroup) and of *F. odoratissimum* representing tropical race 4 (TR4; isolate II-5 originating from Indonesia) (Dita et al. 2011; Maryani et al. 2019), both maintained at the Wageningen University and Research (WUR) collection. The inoculum preparation, disease assays and disease evaluation were performed according to García-Bastidas et al. (2019b). We screened a progeny of 225 genotypes, with five replicates per genotype along with the Mam parent and ‘Gros Michel’ (AAA) as susceptible control for Race 1 and ‘Grand Naine’ as susceptible control for TR4. Seven weeks after inoculation, the disease symptoms of the leaves and the rhizomes were evaluated.

### Heritability of resistance

The heritability (*h*^2^) of resistance to Race 1 was estimated by dividing the genotypic variance ($\sigma_{g}^{2}$) by the sum of the genotypic and environmental variances $\left( {h^{2} = \sigma_{g}^{2} /\left( {\sigma_{g}^{2} + \sigma_{e}^{2} } \right)} \right)$ (Allard 1960). The genotypic variance $\sigma_{g}^{2}$ and the denominator ($\sigma_{g}^{2} + \sigma_{e}^{2}$) were estimated using an ANOVA.

### Genotyping

We collected leaf samples from the segregating population and reference genotypes from either the leaves from plantlets or from the tissue culture plants or from the cigar leaves in the greenhouse. These were lyophilized and used for DNA isolation using the Wizard^®^ Magnetic DNA Purification System for Food kit from (Promega, Madison, USA) following the manufacturer’s instructions. The DNA concentration was quantified using the Quant-iT™ PicoGreen dsDNA Assay Kit (Life Technologies, USA) according to the manufacturer’s instructions. Subsequently, the quantities were calculated using Tecan Infinite^®^ M200 PRO monochromator (Tecan, Männedorf, Switzerland) using Icontrol 107 software (US, Morrisville, NC) and the quality was checked by electrophoresis on 1% agarose gels. The DNA was sent to Diversity Arrays Technology Pty Ltd, Australia, for scoring SNP markers, using the DArTseq platform (http://www.diversityarrays.com/). Based on the DNA sequences flanking the SNPs, the markers were putatively positioned on the genome assembly of Mam DH ‘Pahang’, version 2 (http://banana-genome-hub.southgreen.fr/organism/Musa/acuminata).

Eventually, 217 progeny individuals and their parent were genotyped. The very high number of obtained SNP markers allowed us to apply a very stringent filtering, using as criteria: 1. replicate value = 1; 2. sequences should hit only one position on the reference genome; 3. the polymorphism information content > 0.3; 4. markers should be based on more than ten calls per allele; and 5. we only considered markers that were heterozygous in the parent, and therefore could segregate in the population. We checked the physical position of the filtered, segregating markers on the reference genome, for evaluation of possible homozygous regions in the self-pollinated parent. This subset of qualified markers was used for mapping the Race 1 and TR4 resistance.

### Mapping

Genetic linkage maps were constructed using JoinMap^®^ 6 software with a ‘F2’ population type, thus regarding the heterozygous parent as an F_1_ from a cross between two homozygous grandparents. Markers were assigned to homologous chromosomes, taking linkage or repulsion into consideration. Later, the refined linkage maps were the basis for the mapping analysis. We used MapQTL^®^ 6 to find markers associated with the resistance. The LOD thresholds for significance of quantitative trait locus (QTL) were calculated with a permutation test, using the 95% confidence level and 1000 permutations. Markers above the threshold were supposed to be markers associated with the resistance. We selected genotypes that showed recombination in this region. Subsequently, these recombinants were analysed in detail, applying graphical genotyping, for genetic mapping. This yielded markers flanking the genetic region that contains the resistance gene.

## Results

### Disease assays

**Fig. 1:**
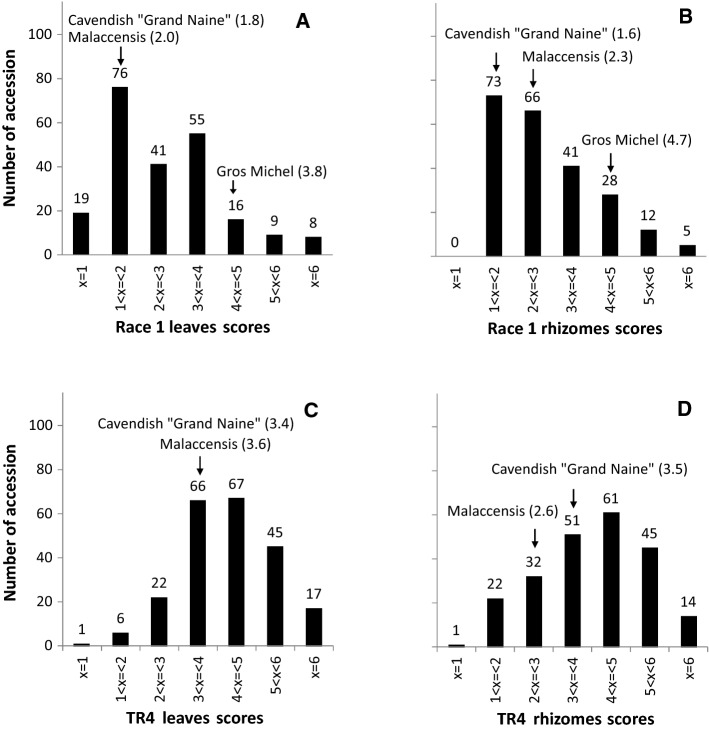
Frequency distribution of disease severity scores for Fusarium wilt in leaves and rhizomes upon inoculations with Race 1 and TR4. *Musa acuminata* ssp. *malaccensis* is the heterozygous parent of the segregating population. Cavendish ‘Grand Naine’ is the control for resistance to Race 1 and for susceptibility to TR4. ‘Gros Michel’ is the susceptible control for Race 1

The histogram of the disease scores of leaves of the segregating population for Race 1 suggests a bimodal distribution of a susceptible group and a relatively resistant group of genotypes, segregating in a 1:1 ratio (Fig. 1a). The 1:1 segregating indicates a monogenic dominant resistance gene that could be heterozygously present in the parent. However, the bimodal distribution was not present for the rhizome symptoms of the same genotypes upon inoculation with Race 1 (Fig. 1b). In contrast to the leaf scores of Race 1, the histograms for disease scores upon inoculation with TR4 showed no bimodal segregation, but rather a normal distribution, both for the leaves and the rhizomes (Fig. 1c, d). Moreover, regarding the leaf symptoms the parent appeared to be more susceptible to TR4 than to Race 1 (Fig. 1c), similar to the response of the susceptible Cavendish ‘Grand Naine’ TR4 check, which was significantly dissimilar from the rhizome scores, where the parent showed a low susceptibility for TR4 (Fig. 1d).

### Heritability of resistance

The heritability (*h*^2^) was calculated to estimate the genetic impact of the resistance to either Race 1 or TR4 in the segregating population. For Race 1, *h*^2^ equalled 0.70 and 0.69 for the leaves and rhizomes, respectively, thus showing very similar levels of heritability of resistance for both plant parts. For TR4, *h*^2^ equalled 0.43 and 0.50 for leaves and rhizomes, respectively. This analysis indicates that the heritability in the resistance of the Mam accession is higher for Race 1 than for TR4, and the genetic impact of TR4 on rhizomes was higher than on the foliage.

### Genotyping

**Fig. 2:**
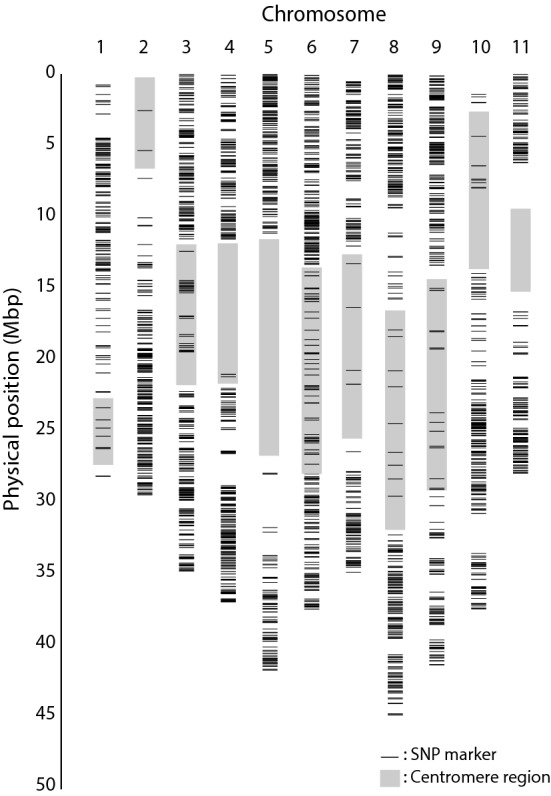
Distribution of SNP markers across the 11 chromosomes according to their physical positions on the reference genome of *Musa acuminata* ssp. *malaccensis* (Mam) DH ‘Pahang’, version 2, released January 2016 (http://banana-genome-hub.southgreen.fr/organism/Musa/acuminata). All chromosomes are covered by segregating markers, which means all chromosomes are heterozygous in the Mam parent that was self-pollinated in this study, thus allowing genetic mapping of all chromosomes in the segregating population

DArTseq provided 32,362 SNP markers for the segregating population. As the population consisted of 225 genotypes, with a few hundred recombinations per chromosome, we did not need that many markers. Therefore, we selected markers based on the above-mentioned very stringent quality criteria, which resulted in 2802 SNP high-quality markers for genetic mapping. All chromosome arms harboured segregating markers, indicating that the parent has been heterozygous for the far majority of the genome (Fig. 2). As DArTseq used a methylation-sensitive restriction enzyme for making the libraries that were sequenced, the DArTseq markers were mainly located in active regions and far less abundant in the centromeric, methylated regions.

**Fig. 3:**
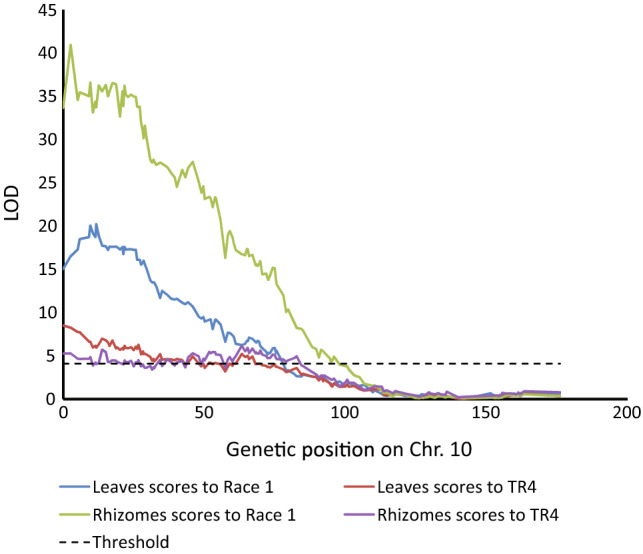
Interval mapping for Fusarium wilt resistance on chromosome 10 to Race 1 and TR4 on *Musa acuminata* ssp. *malaccensis* chromosomes according to leaf and rhizome severity scores. Horizontal lines represent LOD threshold (4.1) value for α = 0.05 after 1000 permutations. Markers above the threshold line indicate a significant association with disease score

We constructed linkage maps according to the selected markers, providing 11 linkage groups (Supplementary Table 1). Subsequently, we performed QTL mapping, using the linkage groups and the phenotypic disease scores for Race 1 and TR4. Only one linkage group (Chromosome 10) showed a significant QTL at the distal part (Fig. 3). Remarkably, this QTL was found for both strains (Race 1 and TR4) and both plant parts (leaves, rhizome). The highest LOD scores were obtained for Race 1 resistance, i.e. 40.9 for the rhizomes and 20.2 for the leaves. For TR4, the maximum LOD scores were far lower, i.e. 5.8 and 5.9 for leaves and rhizomes, respectively.

**Fig. 4:**
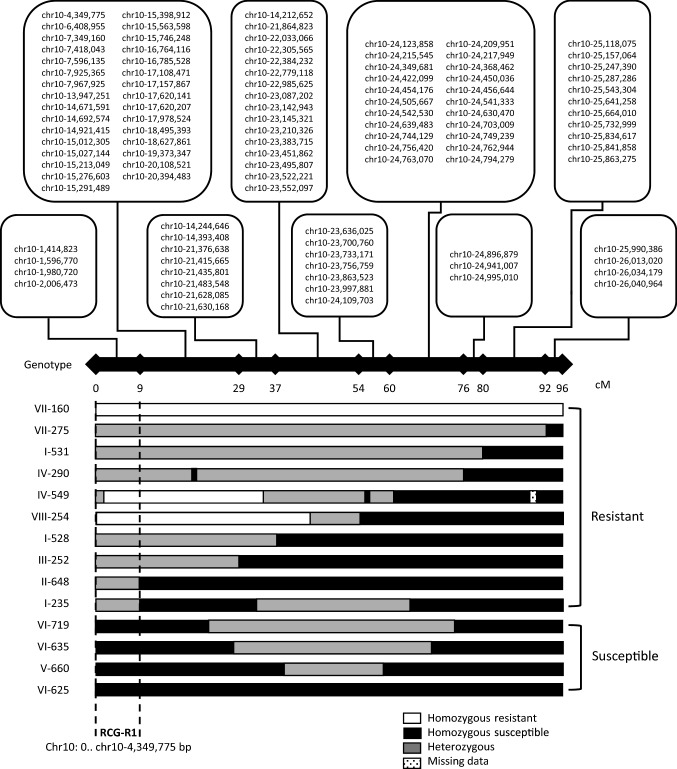
Graphical genotyping of Fusarium wilt resistance to Race 1 on chromosome 10, using rhizomes scores, and DArTseq SNP markers that were significantly associated with resistance. By sorting the selected progeny of selfed *Musa acuminata* ssp. *malaccensis* (Mam) according to the recombination events on chromosome 10 of Mam, the Race 1 resistance could be located in the range of 0–4.3 Mbp at the distal end of the chromosome. The physical positions of the markers on the reference genome (http://banana-genome-hub.southgreen.fr/organism/Musa/acuminata) are shown in the groups of co-segregating markers at the top of the figure

As the combination of Race 1 inoculation and rhizome observations provided the most significant QTL, we focussed on this combination for fine mapping. For this purpose, we selected 13 genotypes that showed recombination in the QTL region and could be clearly designated as either susceptible or resistant regarding the rhizome symptoms. We used 106 SNP markers for depicting the recombination in these genotypes (Fig. 4). According to the physical positions of the markers on the reference genome, the Race 1 resistance is located between 0 and 4.35 Mbp at the distal tip of chromosome 10. This region contains 165 putative genes, and 19 of these putative genes were annotated as leucine-rich repeat (LRR) receptor-like kinase-like genes (Supplementary Table 2).

As shown in Fig. 3, in the same region as the QTL for resistance to Race 1, we found a QTL for TR4 resistance. However, that QTL was far less significant and did not allow fine mapping, due to a less clear phenotypic distinction of susceptible versus resistant genotypes.

We estimated the effect of the presence of the resistance gene on the disease levels upon Race 1 inoculation (Table 1). Homozygous presence of the resistance gene reduced the rhizome symptoms from 4.6 to 2.0, so a reduction of 2.6 on a scale of six classes (Supplementary Figure 1). For leaves, the resistance QTL reduced the symptoms by 1.7. Table 1 shows that for Race 1 the resistance is dominant and also indicates the effect of the presence of the marker associated with Race 1 resistance on resistance to TR4. Although the effect on resistance to TR4 is less pronounced than for Race 1, this table suggests that the resistance for Race 1 might also slightly (reducing the leaves and rhizomes of TR4 symptoms by 1.4, when homozygously present) affect resistance to TR4.

**Table 1:** The mean of disease scores of progeny genotypes lacking the mapped resistance at the distal part of chromosome 10 (aa) or harbouring one copy (ab; heterozygous) or two copies (bb; homozygous) of this resistance QTL

	Rhizomes scores			Leaves scores		
Genotypes	aa	ab	bb	aa	ab	bb
Race 1	2.0 + 0.7	2.6 + 1.0	4.6 + 1.4	2.3 + 1.0	2.3 + 1.0	4.0 + 1.4
TR4	3.4 + 1.3	4.0 + 1.2	4.8 + 1.3	3.4 + 1.1	4.0 + 0.9	4.8 + 0.9

aa: homozygous resistance

ab: heterozygous

bb: homozygous susceptible

## Discussion

In this study, we used resistant Mam originating from Sumatra, Indonesia, which is related to the DH ‘Pahang’, which is resistant to TR4 and whose genome has been sequenced (D’Hont et al. 2012) and is used by many banana researchers as the reference genome for studying resistance against TR4. However, DH ‘Pahang’ has never been used for in mapping studies; thus, there is a possibility that this reference banana may not even have the resistance gene(s) identified in this study. Kayat et al. (2009) used progenies of Mam generated at the University of Malaya for AFLP-based mapping analysis. However, this was unsuccessful due to the limited progeny size. Later, Fraser-Smith et al. (2016) used a selfed population of TR4 resistant and susceptible Mam of unknown origin and concluded that the TR4 resistance is likely under the control of a single gene because the segregation ratio of the number of resistant (R) and susceptible (S) genotypes was 4.67:1 in the first population and 4:1 in the second population. The Mam accession in the current study is resistant to TR4 as well as to Race 1.

In mapping or QTL analyses, molecular markers have been a standard for genetic or linkage maps analysis in many crops (Collard and Mackill 2007). Although genotypic data can utilize various molecular markers, we have chosen DArT markers as they offer a large and cost-effective set of markers from the genome (Kilian et al. 2012). In banana genetics and diversity analysis, DArT was already used and combined with SSR markers to build a linkage map from a segregating progeny of a hybrid between *M. acuminata* ‘Borneo’ and ‘Pisang Lilin’, which resulted in 11 linkage groups (Hippolyte et al. 2010). In this study, we used the SNP markers retrieved from the DArTseq analyses and revealed 11 linkage groups which were used for mapping Fusarium wilt resistance.

Our QTL mapping analysis enabled the mapping of Race 1 resistance on the distal part of chromosome 10 at 0 and 4.35 Mbp. This is the first report of the genetic basis of Race 1 resistance in banana. Previously, genetic analyses did not include mapping studies and hence conclusions were entirely based on the segregation based on phenotypic characters, which do not meet contemporary quality requirements (Vakili 1965; Ssali et al. 2013; Arinaitwe et al. 2019). Our result confirmed that the Race 1 resistance is inherited as a single gene, which accords with Vakili (1965) who used the diploid banana ‘Pisang Lilin’. Our results also indicate that the resistance is controlled by a dominant gene, which contradicts with Ssali et al. (2013) who concluded that the gene was recessively inherited. They used a hybrid population of triploid dessert banana ‘Sukali Ndizi’ (AAB) and a resistant diploid banana ‘TMB2X8075’ (AA). However, this results in a mix of diploid, triploid and tetraploid progeny that complicates adequate analyses due to the complexity of the gametes and the pairing compatibility during the fertilization which may interfere with the proportion of the resistant and the susceptible genotypes (Dodds 1943; Shepherd 1999).

In contrast to Race 1 mapping, we were not able to fine map the TR4 resistance. Although some chromosome 10 markers indicated association with TR4 resistance, the LOD values are low and just significant. This indicated that the population in this study was not well suited for analysing TR4 inheritance in Mam. Nevertheless, we identified a positive interaction between the Race 1 and TR4 resistance loci in the coupling phase (Table 1). A similar observation was reported for two resistance genes (Ph-3 and Sw-5) in coupling phase in tomato resulting in progeny with resistance to tomato spotted wilt virus (TSWV) and *Phytophthora infestans* (the causal agent of late blight in potato and tomato) (Robbins et al. 2010).

As mentioned above, despite the success of Cavendish to manage Fusarium wilt in banana, the genetic basis for resistance to Race 1 remained unclear. Since Race 1 strains are globally disseminated (Ploetz 2015), resistance to Race 1 is a prerequisite for any new banana variety. Developing markers for resistance is, therefore, very valuable as it increases throughput and precision by avoiding cumbersome phenotyping assays, particularly under field conditions. The exceptional and durable nature of Race 1 resistance in Cavendish bananas still requires further study. Our subsequent analyses—19 out of 165 genes are leucine-rich repeat (LRR) receptor-like kinase-like genes—will lead to gene identification and validation, which then can be used to identify whether this gene is also present and expressed and henceforward explains the resistance to Race 1 in Cavendish. It is well established that mutations in LRR domains may predominantly be involved in regulating intramolecular interactions in defence mechanism, i.e. a mutation in the LRR coding region resulted in the loss of nematode resistance in *Nicotiana benthamiana* (Hwang and Williamson 2003). Usually, such mutants result from selection pressure on pathogen populations by deployed resistance factors (Bourguet et al. 2016; Flor 1971). Thus far, however, pathogenic strains on Cavendish bananas exclusively belong to *F. odoratissimum* (García-Bastidas et al. 2019a; Maryani 2018; Ordoñez 2018). This could indicate that the durable resistance to Race 1 in Cavendish is more complicated or that *Fusarium* spp. have alternative strategies to avoid selection pressure by, for example, growing as endophytes in weeds as was recently confirmed by Salacinas (2019). In previous studies, Ordonez et al. (2015) and Maryani et al. (2019) demonstrated that Race 1 comprises several species. Additional studies should reveal whether the identified resistance gene is also effective to the other species in the Race 1 complex.

Thus far, advanced genetic analyses have mostly focused on TR4 (Fraser-Smith et al. 2016; Kayat et al. 2004, 2009; Peraza-Echeverria et al. 2009). Kayat et al. (2009) used AFLP markers, and Fraser-Smith et al. (2016) used a selfed Mam pollination, but both groups never provided mapping data on TR4 resistance. After the preparative studies of Peraza-Echeverria et al. (2008), Dale et al. (2017) cloned *RGA2* which provided resistance to TR4 that was validated by transferring it to the Cavendish cultivar ‘Grand Nain’. This was successfully field-trialled for 3 years and remained free of disease, whereas all checks, including somaclonal variants, succumbed to TR4. Our study enables marker-assisted breeding for Race 1 resistance in banana, which is required in every production environment. Our study also confirms that Mam is fertile and can be a potential parent for breeding Fusarium wilt resistance. Such wild fertile bananas are necessary to create improved diploids that can be used to generate new triploids (Bakry et al. 2009). Additional genetic analyses of other banana accessions with resistance to TR4 should reveal the diversity for the sought-after TR4 resistance across a wide panel of banana germplasm. Taken together, such data will support the breeding of new varieties to manage the threat of FWB to global banana production.

## Electronic supplementary material

Below is the link to the electronic supplementary material.

Supplementary material 1 (DOCX 11 kb)

Supplementary material 2 (PDF 167 kb)
