# Supplementary material for: Genetic mapping of Fusarium wilt resistance in a wild banana Musa acuminata ssp. malaccensis accession
Source: Theor Appl Genet. 2020 Sep 12;133(12):3409–18. doi: 10.1007/s00122-020-03677-y (PMC7567712; doi:10.1007/s00122-020-03677-y)
Supplement: Supplementary file 1 — Supplementary material 1 (DOCX 11 kb) [file 122_2020_3677_MOESM1_ESM.docx]

**Supplementary Figure 1.** Leaves and rhizomes severity scoring according to Garcia-Bastidas et al. (2019b). A. Leaves scores: Score 1 (healthy plant), Score 2 (initial yellowing/ chlorosis), Score 3 (wilting/yellowing leaves ≤10%), D. Score 4 (wilting/yellowing leaves 10%-50%), Score 5 (wilting/yellowing leaves ≤50-%-90%), Score 6 (wilting/yellowing leaves >90% or dead plant). B. Rhizomes scores: Score 1 (No discoloration), Score 2 (≤5% discoloration), Score 3 (6%-10% discoloration), Score 4 (21%-50% discoloration), Score 5 (50-%-90% discoloration), Score 6 (>90% discoloration).

**Supplementary table 1**. Distribution of SNPs marker, physically mapped on the chromosomes of the reference genome of *Musa acuminata* ssp. *malaccensis* DH ‘Pahang’, version 2 (http://banana-genome-hub.southgreen.fr/organism/Musa/acuminata)

**Supplementary table 2**. A list of the predicted genes in the window of the resistance gene for Race 1 according to reference genome of *Musa acuminata* ssp. *malaccensis* DH ‘Pahang’, version 2, (http://banana-genome-hub.southgreen.fr/organism/Musa/acuminata) at the range 0-4.3 Mb.
