# Supplementary material for: Genetic mapping of Fusarium wilt resistance in a wild banana Musa acuminata ssp. malaccensis accession
Source: Theor Appl Genet. 2020 Sep 12;133(12):3409–18. doi: 10.1007/s00122-020-03677-y (PMC7567712; doi:10.1007/s00122-020-03677-y)

Supplementary Table 1.

Chromosome	Total SNP markers	Filtered SNPs markers	Lenght of genetic distance (cM)
1	1,870	162	258
2	1,973	229	165
3	2,664	328	281
4	3,088	305	207
5	2,283	254	258
6	3,021	325	302
7	2,204	224	221
8	2,732	303	256
9	2,617	277	254
10	2,441	208	440
11	1,818	187	186
Mitochondria	313		
Unanchored	5,338		
Total	32,362	2,802	2,828
Mean	2,428	255	257

Supplementary Table 2.

Gene code	Physical position in the DH "Pahang"		Putative gene
	start	stop	
Ma10_p00010.1	106217	108235	Ma10_g00010~ exocyst complex component EXO70A1-like~ unknown_gene~ missing_functional_completeness
Ma10_p00020.1	516157	519871	Ma10_g00020~ craniofacial development protein 1-like~ unknown_gene~ missing_functional_completeness
Ma10_p00030.1	597395	606009	Ma10_g00030~ endoplasmic reticulum-Golgi intermediate compartment protein 3-like~ unknown_gene~ missing_functional_completenes
Ma10_p00040.1	626059	628374	Ma10_g00040~ uncharacterized glycosyl hydrolase Rv2006/MT2062, putative, expressed~ Rv2006~ complete
Ma10_p00050.1	752579	753205	Ma10_g00050~ mavicyanin-like~ unknown_gene~ missing_functional_completeness
Ma10_p00060.1	754512	778558	Ma10_g00060~ coiled-coil domain-containing protein 22 homolog, transcript variant X1~ unknown_gene~ missing_functional_completeness
Ma10_p00060.2	754512	778558	Ma10_g00060~ coiled-coil domain-containing protein 22 homolog, transcript variant X1~ unknown_gene~ missing_functional_completeness
Ma10_p00060.3	754512	778558	Ma10_g00060~ coiled-coil domain-containing protein 22 homolog, transcript variant X1~ unknown_gene~ missing_functional_completeness
Ma10_p00060.4	754512	778558	Ma10_g00060~ coiled-coil domain-containing protein 22 homolog, transcript variant X1~ unknown_gene~ missing_functional_completeness
Ma10_p00070.1	909174	911817	Ma10_g00070~ serine/threonine-protein kinase D6PKL1-like, transcript variant X1~ unknown_gene~ missing_functional_completeness
Ma10_p00070.2	909174	911817	Ma10_g00070~ serine/threonine-protein kinase D6PKL1-like, transcript variant X1~ unknown_gene~ missing_functional_completeness
Ma10_p00070.3	909174	911817	Ma10_g00070~ serine/threonine-protein kinase D6PKL1-like, transcript variant X1~ unknown_gene~ missing_functional_completeness
Ma10_p00080.1	913616	914370	Ma10_g00080~ UDP-glycosyltransferase 89A2-like~ unknown_gene~ missing_functional_completeness
Ma10_p00090.1	1076495	1079259	Ma10_g00090~ transmembrane protein 245-like~ unknown_gene~ missing_functional_completeness
Ma10_p00100.3	1251994	1260969	Ma10_g00100~ formin-binding protein 4, transcript variant X1~ unknown_gene~ missing_functional_completeness
Ma10_p00100.1	1255854	1260969	Ma10_g00100~ formin-binding protein 4, transcript variant X1~ unknown_gene~ missing_functional_completeness
Ma10_p00100.2	1255854	1260969	Ma10_g00100~ formin-binding protein 4, transcript variant X1~ unknown_gene~ missing_functional_completeness
Ma10_p00110.1	1261682	1271748	Ma10_g00110~ calcineurin B-like protein 3~ unknown_gene~ missing_functional_completeness
Ma10_p00120.1	1347091	1349143	Ma10_g00120~ glucan endo-1,3-beta-glucosidase 14-like, transcript variant X1~ unknown_gene~ missing_functional_completeness
Ma10_p00120.2	1347091	1348198	Ma10_g00120~ glucan endo-1,3-beta-glucosidase 14-like, transcript variant X1~ unknown_gene~ missing_functional_completeness
Ma10_p00130.1	1352125	1355477	Ma10_g00130~ calcium-binding mitochondrial carrier protein SCaMC-1-like~ unknown_gene~ missing_functional_completeness
Ma10_p00140.1	1380269	1381801	Ma10_g00140~ 3-ketoacyl-CoA synthase 11~ KCS5~ complete
Ma10_p00150.1	1383864	1387312	Ma10_g00150~ zeaxanthin epoxidase, chloroplastic~ unknown_gene~ missing_functional_completeness
Ma10_p00160.1	1387497	1389440	Ma10_g00160~ pentatricopeptide repeat-containing protein At1g31430~ unknown_gene~ missing_functional_completeness
Ma10_p00170.1	1392114	1395221	Ma10_g00170~ root phototropism protein 3-like~ unknown_gene~ missing_functional_completeness
Ma10_p00180.1	1408918	1415692	Ma10_g00180~ putative pleiotropic drug resistance protein 7~ unknown_gene~ missing_functional_completeness
Ma10_p00190.1	1418732	1434365	Ma10_g00190~ coiled-coil domain-containing protein 93, transcript variant X1~ unknown_gene~ missing_functional_completeness
Ma10_p00190.2	1418732	1434365	Ma10_g00190~ coiled-coil domain-containing protein 93, transcript variant X1~ unknown_gene~ missing_functional_completeness
Ma10_p00200.1	1436216	1437394	Ma10_g00200~ gibberellin 3-beta-dioxygenase 1-like~ unknown_gene~ missing_functional_completeness
Ma10_p00210.1	1455447	1457078	Ma10_g00210~ F-box/kelch-repeat protein At1g80440-like~ unknown_gene~ missing_functional_completeness
Ma10_p00220.1	1457117	1458548	Ma10_g00220~ uncharacterized LOC103973058, transcript variant X3~ unknown_gene~ missing_functional_completeness
Ma10_p00220.2	1457117	1458548	Ma10_g00220~ uncharacterized LOC103973058, transcript variant X3~ unknown_gene~ missing_functional_completeness
Ma10_p00220.3	1457117	1458548	Ma10_g00220~ uncharacterized LOC103973058, transcript variant X3~ unknown_gene~ missing_functional_completeness
Ma10_p00230.1	1462268	1462450	Ma10_g00230~ Putative Arabinogalactan peptide 22~ AGP22~ modules
Ma10_p00240.1	1464833	1465264	Ma10_g00240~ uncharacterized LOC103973057~ unknown_gene~ missing_functional_completeness
Ma10_p00250.1	1491520	1498629	Ma10_g00250~ uncharacterized LOC103973056, transcript variant X2~ unknown_gene~ missing_functional_completeness
Ma10_p00250.2	1492130	1498629	Ma10_g00250~ uncharacterized LOC103973056, transcript variant X2~ unknown_gene~ missing_functional_completeness
Ma10_p00260.1	1499455	1502720	Ma10_g00260~ UDP-galactose/UDP-glucose transporter 3-like~ unknown_gene~ missing_functional_completeness
Ma10_p00270.1	1521833	1522366	Ma10_g00270~ PRA1 family protein F3-like~ unknown_gene~ missing_functional_completeness
Ma10_p00280.1	1524018	1526418	Ma10_g00280~ putative transcription factor bHLH041~ unknown_gene~ missing_functional_completeness
Ma10_p00290.1	1527067	1531164	Ma10_g00290~ Putative Transmembrane 9 superfamily member 4~ TM9SF4~ complete
Ma10_p00300.1	1539387	1541792	Ma10_g00300~ eukaryotic initiation factor 4A-15-like~ unknown_gene~ missing_functional_completeness
Ma10_p00310.1	1550418	1551008	Ma10_g00310~ uncharacterized LOC103973081~ unknown_gene~ missing_functional_completeness
Ma10_p00320.1	1552114	1553667	Ma10_g00320~ zinc transporter 7-like~ unknown_gene~ missing_functional_completeness
Ma10_p00330.1	1554631	1559684	Ma10_g00330~ reticulocalbin-2-like~ unknown_gene~ missing_functional_completeness
Ma10_p00340.1	1573871	1575950	Ma10_g00340~ zinc finger protein NUTCRACKER-like, transcript variant X1~ unknown_gene~ missing_functional_completeness
Ma10_p00340.2	1573871	1575950	Ma10_g00340~ zinc finger protein NUTCRACKER-like, transcript variant X1~ unknown_gene~ missing_functional_completeness
Ma10_p00340.3	1573871	1575950	Ma10_g00340~ zinc finger protein NUTCRACKER-like, transcript variant X1~ unknown_gene~ missing_functional_completeness
Ma10_p00350.1	1579421	1589344	Ma10_g00350~ far upstream element-binding protein 2-like~ unknown_gene~ missing_functional_completeness
Ma10_p00360.1	1589916	1590766	Ma10_g00360~ inorganic pyrophosphatase 2-like, transcript variant X1~ unknown_gene~ missing_functional_completeness
Ma10_p00360.2	1589916	1590997	Ma10_g00360~ inorganic pyrophosphatase 2-like, transcript variant X1~ unknown_gene~ missing_functional_completeness
Ma10_p00370.1	1593942	1595180	Ma10_g00370~ probable F-box protein At4g22030~ unknown_gene~ missing_functional_completeness
Ma10_p00380.1	1600629	1604047	Ma10_g00380~ Putative Whole genome shotgun sequence of line PN40024, scaffold_8.assembly12x (Fragment)~ ATH1~ modules
Ma10_p00390.1	1606305	1607438	Ma10_g00390~ zinc finger CCCH domain-containing protein 35-like~ unknown_gene~ missing_functional_completeness
Ma10_p00400.1	1614199	1615123	Ma10_g00400~ probable LRR receptor-like serine/threonine-protein kinase At1g51820~ unknown_gene~ missing_functional_completeness
Ma10_p00410.1	1618250	1621530	Ma10_g00410~ Putative Probable LRR receptor-like serine/threonine-protein kinase At1g05700~ At1g05700~ fragment
Ma10_p00420.1	1628209	1629339	Ma10_g00420~ LRR receptor-like serine/threonine-protein kinase ERECTA~ unknown_gene~ missing_functional_completeness

Ma10_p00430.1	1651242	1652705	Ma10_g00430~ Probable LRR receptor-like serine/threonine-protein kinase At1g51820~ At1g51820~ fragment
Ma10_p00440.1	1658083	1658712	Ma10_g00440~ probably inactive leucine-rich repeat receptor-like protein kinase At2g25790~ unknown_gene~ missing_functional_completeness
Ma10_p00450.1	1671933	1679978	Ma10_g00450~ Putative Probable LRR receptor-like serine/threonine-protein kinase At4g36180~ At4g36180~ complete
Ma10_p00460.1	1681347	1682052	Ma10_g00460~ Hypothetical protein~ At1g51820~ missing_functional_completeness; Probable LRR receptor-like serine/threonine-protei kinase At1g05700; PREDICTED: probable LRR receptor-like serine/threonine-protein kinase At4g29180 isoform X4 [Musa acuminata subsp. malaccensis]
Ma10_p00470.1	1684067	1686962	Ma10_g00470~ leucine rich repeat protein, putative~ ERL1~ complete
Ma10_p00480.1	1692198	1694093	Ma10_g00480~ probable LRR receptor-like serine/threonine-protein kinase At4g36180~ unknown_gene~ missing_functional_completeness
Ma10_p00490.1	1710726	1713763	Ma10_g00490~ Putative Probable LRR receptor-like serine/threonine-protein kinase At4g36180~ At4g36180~ complete
Ma10_p00500.1	1752896	1754926	Ma10_g00500~ leucine-rich repeat receptor-like protein kinase PEPR2~ unknown_gene~ missing_functional_completeness
Ma10_p00510.1	1754955	1761490	Ma10_g00510~ LRR receptor-like serine/threonine-protein kinase ERECTA~ unknown_gene~ missing_functional_completeness
Ma10_p00520.1	1766856	1768436	Ma10_g00520~ Putative Probable LRR receptor-like serine/threonine-protein kinase At1g05700~ At1g05700~ fragment
Ma10_p00530.1	1768529	1769229	Ma10_g00530~ Hypothetical protein~ PGIP3~ missing_functional_completeness; PREDICTED: LRR receptor-like serine/threonine-protei kinase FLS2 [Musa acuminata subsp. malaccensis] Select seq ref|XP_009385819.1|
Ma10_p00540.1	1779420	1781023	Ma10_g00540~ Hypothetical protein~ unknown_gene~ missing_functional_completeness
Ma10_p00550.1	1781038	1783746	Ma10_g00550~ probable leucine-rich repeat receptor-like protein kinase At1g35710~ unknown_gene~ missing_functional_completeness
Ma10_p00560.1	1785620	1786664	Ma10_g00560~ leucine-rich repeat receptor-like protein CLAVATA2~ unknown_gene~ missing_functional_completeness
Ma10_p00570.1	1814769	1817522	Ma10_g00570~ LRR receptor-like serine/threonine-protein kinase FLS2~ unknown_gene~ missing_functional_completeness
Ma10_p00580.1	1818828	1818959	Ma10_g00580~ Hypothetical protein~ slc17a6b~ missing_functional_completeness
Ma10_p00590.1	1847632	1849029	Ma10_g00590~ protein IQ-DOMAIN 14-like, transcript variant X1~ unknown_gene~ missing_functional_completeness
Ma10_p00590.2	1847632	1849029	Ma10_g00590~ protein IQ-DOMAIN 14-like, transcript variant X1~ unknown_gene~ missing_functional_completeness
Ma10_p00600.1	1865930	1868792	Ma10_g00600~ auxin-responsive protein IAA16-like~ unknown_gene~ missing_functional_completeness
Ma10_p00610.1	1875192	1879331	Ma10_g00610~ 40S ribosomal protein S24-1-like~ unknown_gene~ missing_functional_completeness
Ma10_p00620.1	1882093	1886270	Ma10_g00620~ transcription factor MYB1R1-like~ unknown_gene~ missing_functional_completeness
Ma10_p00630.1	1887218	1921335	Ma10_g00630~ probable RNA-dependent RNA polymerase 5, transcript variant X2~ unknown_gene~ missing_functional_completeness
Ma10_p00630.2	1887218	1918276	Ma10_g00630~ probable RNA-dependent RNA polymerase 5, transcript variant X2~ unknown_gene~ missing_functional_completeness
Ma10_p00630.3	1887218	1921335	Ma10_g00630~ probable RNA-dependent RNA polymerase 5, transcript variant X2~ unknown_gene~ missing_functional_completeness
Ma10_p00630.4	1887218	1921335	Ma10_g00630~ probable RNA-dependent RNA polymerase 5, transcript variant X2~ unknown_gene~ missing_functional_completeness
Ma10_p00640.1	1926276	1928030	Ma10_g00640~ uncharacterized LOC103973075~ unknown_gene~ missing_functional_completeness
Ma10_p00650.1	1954630	1956555	Ma10_g00650~ LRR receptor-like serine/threonine-protein kinase GSO1~ unknown_gene~ missing_functional_completeness
Ma10_p00660.1	1956649	1957080	Ma10_g00660~ receptor-like protein 2~ unknown_gene~ missing_functional_completeness; PREDICTED: leucine-rich repeat receptor protein kinase MSL1-like [Musa acuminata subsp. malaccensis] Select seq ref|XP_009385818.1|
Ma10_p00670.1	1970155	1970781	Ma10_g00670~ uncharacterized LOC103973034~ unknown_gene~ missing_functional_completeness
Ma10_p00680.1	1977712	1979532	Ma10_g00680~ uncharacterized protein DDB_G0290685-like, transcript variant X2~ unknown_gene~ missing_functional_completeness
Ma10_p00680.2	1977712	1979532	Ma10_g00680~ uncharacterized protein DDB_G0290685-like, transcript variant X2~ unknown_gene~ missing_functional_completeness
Ma10_p00690.1	1980557	1983954	Ma10_g00690~ monoglyceride lipase-like~ unknown_gene~ missing_functional_completeness
Ma10_p00700.1	1984946	1992137	Ma10_g00700~ phosphoribosylaminoimidazole carboxylase, chloroplastic-like, transcript variant X2~ unknown_gene~ missing_functional_completeness
Ma10_p00700.2	1987221	1992137	Ma10_g00700~ phosphoribosylaminoimidazole carboxylase, chloroplastic-like, transcript variant X2~ unknown_gene~ missing_functional_completeness
Ma10_p00710.1	1994710	2012932	Ma10_g00710~ vacuolar fusion protein CCZ1 homolog, transcript variant X1~ unknown_gene~ missing_functional_completeness
Ma10_p00710.2	1994710	2012932	Ma10_g00710~ vacuolar fusion protein CCZ1 homolog, transcript variant X1~ unknown_gene~ missing_functional_completeness
Ma10_p00720.1	2016911	2017750	Ma10_g00720~ zinc-finger homeodomain protein 2-like~ unknown_gene~ missing_functional_completeness
Ma10_p00730.1	2146537	2147660	Ma10_g00730~ glutaredoxin domain containing protein, putative, expressed~ hpaP~ fragment
Ma10_p00740.1	2156552	2156758	Ma10_g00740~ Protein EPIDERMAL PATTERNING FACTOR 2~ EPF2~ fragment
Ma10_p00750.1	2398024	2406142	Ma10_g00750~ nascent polypeptide-associated complex subunit alpha-like protein 1~ unknown_gene~ missing_functional_completeness
Ma10_p00760.1	2407385	2408898	Ma10_g00760~ Lichenase~ GNS1~ complete
Ma10_p00770.1	2479475	2479585	Ma10_g00770~ Acyl carrier protein, mitochondrial~ At2g44620~ fragment
Ma10_p00780.1	2653315	2654362	Ma10_g00780~ Hypothetical protein~ unknown_gene~ missing_functional_completeness
Ma10_p00790.1	2654454	2658109	Ma10_g00790~ adenosine deaminase-like protein, transcript variant X2~ unknown_gene~ missing_functional_completeness
Ma10_p00790.2	2654454	2658173	Ma10_g00790~ adenosine deaminase-like protein, transcript variant X2~ unknown_gene~ missing_functional_completeness
Ma10_p00800.1	2713357	2713455	Ma10_g00800~ Elongation factor G~ fusA~ fragment
Ma10_p00810.1	3063251	3065332	Ma10_g00810~ regulatory protein NPR5-like, transcript variant X2~ unknown_gene~ missing_functional_completeness
Ma10_p00810.2	3063251	3065332	Ma10_g00810~ regulatory protein NPR5-like, transcript variant X2~ unknown_gene~ missing_functional_completeness
Ma10_p00820.1	3443968	3445788	Ma10_g00820~ putative pentatricopeptide repeat-containing protein At1g56570~ unknown_gene~ missing_functional_completeness
Ma10_p00830.1	3645541	3711155	Ma10_g00830~ uncharacterized LOC103999822~ unknown_gene~ missing_functional_completeness
Ma10_p00840.1	3752896	3765321	Ma10_g00840~ RRP12-like protein~ unknown_gene~ missing_functional_completeness
Ma10_p00850.1	3781738	3782094	Ma10_g00850~ Putative LTPL2 - Protease inhibitor/seed storage/LTP family protein precursor, expressed~ ZK686.2~ fragment
Ma10_p00860.1	3790199	3792038	Ma10_g00860~ neurogenic protein mastermind-like~ unknown_gene~ missing_functional_completeness
Ma10_p00870.1	3799888	3800676	Ma10_g00870~ Hypothetical protein~ uba3~ missing_functional_completeness
Ma10_p00880.1	3805682	3810351	Ma10_g00880~ DNA repair protein UVH3~ UVH3~ fragment
Ma10_p00890.1	3816940	3819301	Ma10_g00890~ ARM REPEAT PROTEIN INTERACTING WITH ABF2-like~ unknown_gene~ missing_functional_completeness
Ma10_p00900.1	3832611	3833202	Ma10_g00900~ uncharacterized LOC104000068~ unknown_gene~ missing_functional_completeness
Ma10_p00910.1	3835987	3854439	Ma10_g00910~ DNA repair protein UVH3, transcript variant X1~ unknown_gene~ missing_functional_completeness

Ma10_p00910.2	3835987	3854439	Ma10_g00910~ DNA repair protein UVH3, transcript variant X1~ unknown_gene~ missing_functional_completeness
Ma10_p00910.3	3835987	3854439	Ma10_g00910~ DNA repair protein UVH3, transcript variant X1~ unknown_gene~ missing_functional_completeness
Ma10_p00910.4	3835987	3854439	Ma10_g00910~ DNA repair protein UVH3, transcript variant X1~ unknown_gene~ missing_functional_completeness
Ma10_p00920.1	3856484	3861715	Ma10_g00920~ Hypothetical protein~ RPS3C~ missing_functional_completeness
Ma10_p00930.1	3861722	3862640	Ma10_g00930~ Hypothetical protein~ unknown_gene~ missing_functional_completeness
Ma10_p00940.1	3873099	3873409	Ma10_g00940~ Putative Uncharacterized protein 126R~ IIV3-126R~ fragment
Ma10_p00950.1	3873577	3875723	Ma10_g00950~ Putative Ankyrin repeat and BTB/POZ domain-containing protein 2~ ABTB2~ fragment
Ma10_p00960.1	3875676	3878833	Ma10_g00960~ ARM REPEAT PROTEIN INTERACTING WITH ABF2-like~ unknown_gene~ missing_functional_completeness
Ma10_p00970.1	3881030	3881509	Ma10_g00970~ Hypothetical protein~ unknown_gene~ missing_functional_completeness
Ma10_p01060.1	3881062	3941845	Ma10_g01060~ ARM REPEAT PROTEIN INTERACTING WITH ABF2-like~ unknown_gene~ missing_functional_completeness
Ma10_p00980.1	3890937	3894777	Ma10_g00980~ ABTB1 - Armadillo repeats with a Bric-a-Brac, Tramtrack, Broad Complex BTB domain, expressed~ unknown_gene~ fragment
Ma10_p00990.1	3896508	3896748	Ma10_g00990~ DNA repair protein UVH3~ UVH3~ fragment
Ma10_p01000.1	3898518	3898882	Ma10_g01000~ DNA repair protein UVH3-like~ unknown_gene~ missing_functional_completeness
Ma10_p01010.1	3902908	3903290	Ma10_g01010~ Hypothetical protein~ unknown_gene~ missing_functional_completeness
Ma10_p01020.1	3904111	3909869	Ma10_g01020~ ARM REPEAT PROTEIN INTERACTING WITH ABF2-like~ unknown_gene~ missing_functional_completeness
Ma10_p01030.1	3918399	3919134	Ma10_g01030~ DNA repair protein UVH3~ UVH3~ fragment
Ma10_p01040.1	3925690	3929958	Ma10_g01040~ ABTB1 - Armadillo repeats with a Bric-a-Brac, Tramtrack, Broad Complex BTB domain, expressed~ VAC8~ fragment
Ma10_p01050.1	3934264	3935047	Ma10_g01050~ ARM REPEAT PROTEIN INTERACTING WITH ABF2-like~ unknown_gene~ missing_functional_completeness
Ma10_p01070.1	3944788	3949487	Ma10_g01070~ peroxisomal adenine nucleotide carrier 1-like~ unknown_gene~ missing_functional_completeness
Ma10_p01080.1	3952495	3988186	Ma10_g01080~ potassium transporter 7-like~ unknown_gene~ missing_functional_completeness
Ma10_p01090.1	3990712	3994653	Ma10_g01090~ serine/threonine-protein kinase PBS1-like~ unknown_gene~ missing_functional_completeness
Ma10_p01100.1	3996682	4006881	Ma10_g01100~ sodium/hydrogen exchanger 2-like~ unknown_gene~ missing_functional_completeness
Ma10_p01110.1	4010551	4019481	Ma10_g01110~ vesicle-associated protein 1-2-like, transcript variant X2~ unknown_gene~ missing_functional_completeness
Ma10_p01110.2	4010551	4019481	Ma10_g01110~ vesicle-associated protein 1-2-like, transcript variant X2~ unknown_gene~ missing_functional_completeness
Ma10_p01120.1	4023728	4025648	Ma10_g01120~ secoisolariciresinol dehydrogenase-like, transcript variant X1~ unknown_gene~ missing_functional_completeness
Ma10_p01120.2	4023728	4025648	Ma10_g01120~ secoisolariciresinol dehydrogenase-like, transcript variant X1~ unknown_gene~ missing_functional_completeness
Ma10_p01130.1	4027514	4031525	Ma10_g01130~ 1-aminocyclopropane-1-carboxylate oxidase homolog 3-like~ unknown_gene~ missing_functional_completeness
Ma10_p01140.1	4041851	4068539	Ma10_g01140~ histone-lysine N-methyltransferase setd3~ unknown_gene~ missing_functional_completeness
Ma10_p01150.1	4074177	4110993	Ma10_g01150~ protein NRDE2 homolog~ unknown_gene~ missing_functional_completeness
Ma10_p01160.1	4114992	4116311	Ma10_g01160~ probable anion transporter 6, transcript variant X2~ unknown_gene~ missing_functional_completeness
Ma10_p01160.2	4114992	4116311	Ma10_g01160~ probable anion transporter 6, transcript variant X2~ unknown_gene~ missing_functional_completeness
Ma10_p01170.1	4126875	4129467	Ma10_g01170~ gamma-tubulin complex component 3-like~ unknown_gene~ missing_functional_completeness
Ma10_p01180.1	4159472	4161513	Ma10_g01180~ protein TRANSPARENT TESTA 1-like~ unknown_gene~ missing_functional_completeness
Ma10_p01190.1	4240738	4241247	Ma10_g01190~ uncharacterized LOC103999870~ unknown_gene~ missing_functional_completeness
Ma10_p01200.1	4242611	4243120	Ma10_g01200~ Hypothetical protein~ FAM129C~ missing_functional_completeness
Ma10_p01210.1	4246700	4247011	Ma10_g01210~ histone H4~ unknown_gene~ missing_functional_completeness
Ma10_p01220.1	4248690	4253026	Ma10_g01220~ sporulation-specific protein 15-like~ unknown_gene~ missing_functional_completeness
Ma10_p01230.1	4284105	4285001	Ma10_g01230~ protein SENSITIVITY TO RED LIGHT REDUCED 1, transcript variant X1~ unknown_gene~ missing_functional_completeness
Ma10_p01230.2	4284105	4285001	Ma10_g01230~ protein SENSITIVITY TO RED LIGHT REDUCED 1, transcript variant X1~ unknown_gene~ missing_functional_completeness
Ma10_p01240.1	4293509	4297526	Ma10_g01240~ uncharacterized LOC103999871~ unknown_gene~ missing_functional_completeness
Ma10_p01250.1	4320226	4323566	Ma10_g01250~ protein IN2-1 homolog B-like~ unknown_gene~ missing_functional_completeness
Ma10_p01260.1	4330518	4333327	Ma10_g01260~ serine/threonine-protein kinase PBS1-like, transcript variant X2~ unknown_gene~ missing_functional_completeness
Ma10_p01260.2	4330518	4333327	Ma10_g01260~ serine/threonine-protein kinase PBS1-like, transcript variant X2~ unknown_gene~ missing_functional_completeness
Ma10_p01270.1	4334006	4340804	Ma10_g01270~ elongator complex protein 6, transcript variant X2~ unknown_gene~ missing_functional_completeness
Ma10_p01270.2	4334006	4340804	Ma10_g01270~ elongator complex protein 6, transcript variant X2~ unknown_gene~ missing_functional_completeness
Ma10_p01270.3	4334006	4340804	Ma10_g01270~ elongator complex protein 6, transcript variant X2~ unknown_gene~ missing_functional_completeness
Ma10_p01280.1	4347745	4351833	Ma10_g01280~ AP2-like ethylene-responsive transcription factor TOE3, transcript variant X3~ unknown_gene~ missing_functional_completeness
Ma10_p01280.2	4347745	4351833	Ma10_g01280~ AP2-like ethylene-responsive transcription factor TOE3, transcript variant X3~ unknown_gene~ missing_functional_completeness
Ma10_p01280.3	4347745	4351833	Ma10_g01280~ AP2-like ethylene-responsive transcription factor TOE3, transcript variant X3~ unknown_gene~ missing_functional_completeness
Ma10_p01290.1	4396454	4397485	Ma10_g01290~ uncharacterized LOC103999867~ unknown_gene~ missing_functional_completeness

*Highlighted: putative resistance gene analogue

Supplementary Figure 1

A. Leaves scores

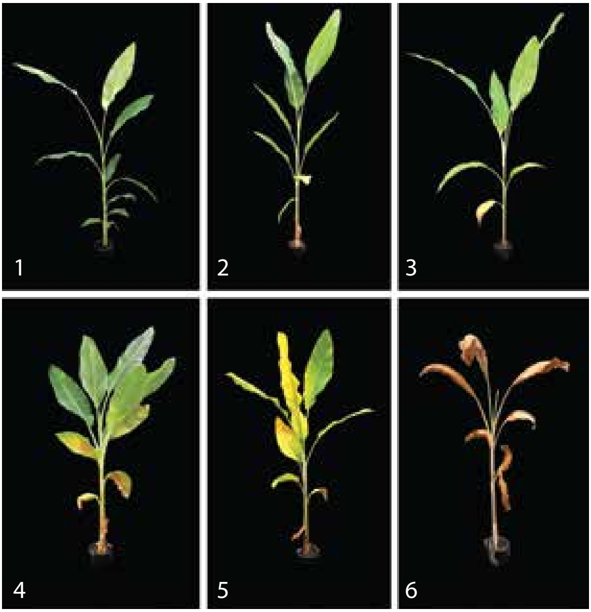

B. Rhizomes scores

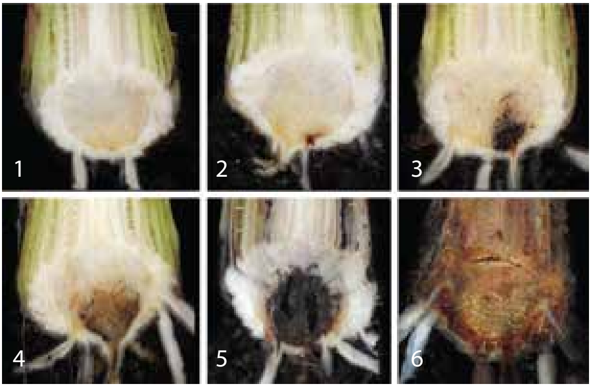

Supplement: Supplementary file 2 — Supplementary material 2 (PDF 167 kb) [file 122_2020_3677_MOESM2_ESM.pdf]
